# Post-traumatic liver injury caused by chest tube placement: myths and controversies in conservative management

**DOI:** 10.1093/jscr/rjag372

**Published:** 2026-05-19

**Authors:** David S Álvarez Gahona, Erika D Montenegro Garcia, Joseph D Valdivieso Vélez, Erick G Chiriboga Bombón, David I Narváez Salas, Fernando I Zumárraga López, María F Piedra Cevallos, Valeria A Rivera Rodríguez

**Affiliations:** Department of General Surgery Hospital de Especialidades Eugenio Espejo, Pontificia Universidad Católica del Ecuador, Av. Gran Colombia s/n y Yaguachi, 170403 Quito, Ecuador; Pontificia Universidad Católica del Ecuador, Department of General Surgery Hospital Padre Carollo, Av. Rumichaca S33-10, 170702 Quito, Ecuador; Pontificia Universidad Católica del Ecuador, Department of General Surgery Hospital Northospital, Av. de la Prensa, 170104 Quito, Ecuador; Department of General Surgery Hospital de Especialidades Eugenio Espejo, Pontificia Universidad Católica del Ecuador, Av. Gran Colombia s/n y Yaguachi, 170403 Quito, Ecuador; Department of General Surgery Hospital Vozandes Quito, Av. Juan José de Villalengua Oe2-37, 170521 Quito, Ecuador; Pontificia Universidad Católica del Ecuador, Department of General Surgery Hospital de Especialidades Carlos Andrade Marín, Av. Ayacucho N19-63, 170103 Quito, Ecuador; Universidad de las Américas, Av. de los Granados E12-41, 170513 Quito, Ecuador; Department of General Surgery Hospital de Especialidades Eugenio Espejo, Pontificia Universidad Católica del Ecuador, Av. Gran Colombia s/n y Yaguachi, 170403 Quito, Ecuador

**Keywords:** chest tube, trauma, conservative management, iatrogenic injury, liver trauma

## Abstract

This case report describes the conservative management of an iatrogenic hepatic injury secondary to chest tube thoracostomy in a 54-year-old male with severe thoracic polytrauma. A right-sided chest tube inserted in the seventh intercostal space for hemothorax resulted in transhepatic malposition. Given hemodynamic stability and absence of active hemorrhage, non-operative management (device removal and primary suture) was undertaken with strict monitoring and favorable evolution. This case underscores the feasibility of conservative management in selected patients and cautions against automatic classification as impalement trauma requiring laparotomy.

## Introduction

Chest tube thoracostomy is a fundamental procedure in emergency and surgical practice [1, 2]. Its principal indication in pneumothorax, as well as hemothorax or significant pleural effusion secondary to thoracic trauma. Over 50% of trauma patients in emergency departments present with thoracic injuries requiring pleural drainage [3].

Complication rates range from 3.4% to 36% [4]. The most frequent adverse events include thoracic malposition (intraparenchymal, intercostal, mediastinal) [5], abdominal malposition, and infectious complications. Injury to intra-abdominal viscera, stomach, spleen, and liver [3] is rare but potentially life-threatening [5]. Reports of chest tubes traversing hepatic parenchyma into the inferior vena cava or causing portal vein injury are exceptional [6, 7].

Penetrating hepatic trauma secondary to pleural tube placement represents an uncommon subset of liver injury. Mortality has decreased to 4–15% with advances in trauma care, and non-operative management (NOM), extrapolated from blunt hepatic trauma protocols, has demonstrated effectiveness [4].

We report a case of iatrogenic liver injury secondary to chest tube placement for pleural drainage that was successfully managed using a conservative approach.

## Case report

A 54-year-old male without comorbidities sustained thoracoabdominal crush trauma when a parked bus fell onto his neck, chest, and abdomen. He remained conscious and was initially managed at a secondary level hospital. Primary survey revealed asymmetric chest expansion, depression of the left hemithorax, bilateral clavicular deformity, cervicothoracic subcutaneous emphysema, and palpable crepitus. Breath sounds were decreased bilaterally. The abdomen was soft, depressible, and without peritoneal signs.

Imaging demonstrated bilateral pneumothorax and hemothorax (left predominance), non-displaced fracture of the middle third of the sternum, bilateral clavicle fractures, and left rib fractures (second displaced; third to fifth). Bilateral thoracostomies drained 500 mL hematic fluid on the right and 50 mL on the left, with incomplete pneumothorax resolution.

**Figure 1:**
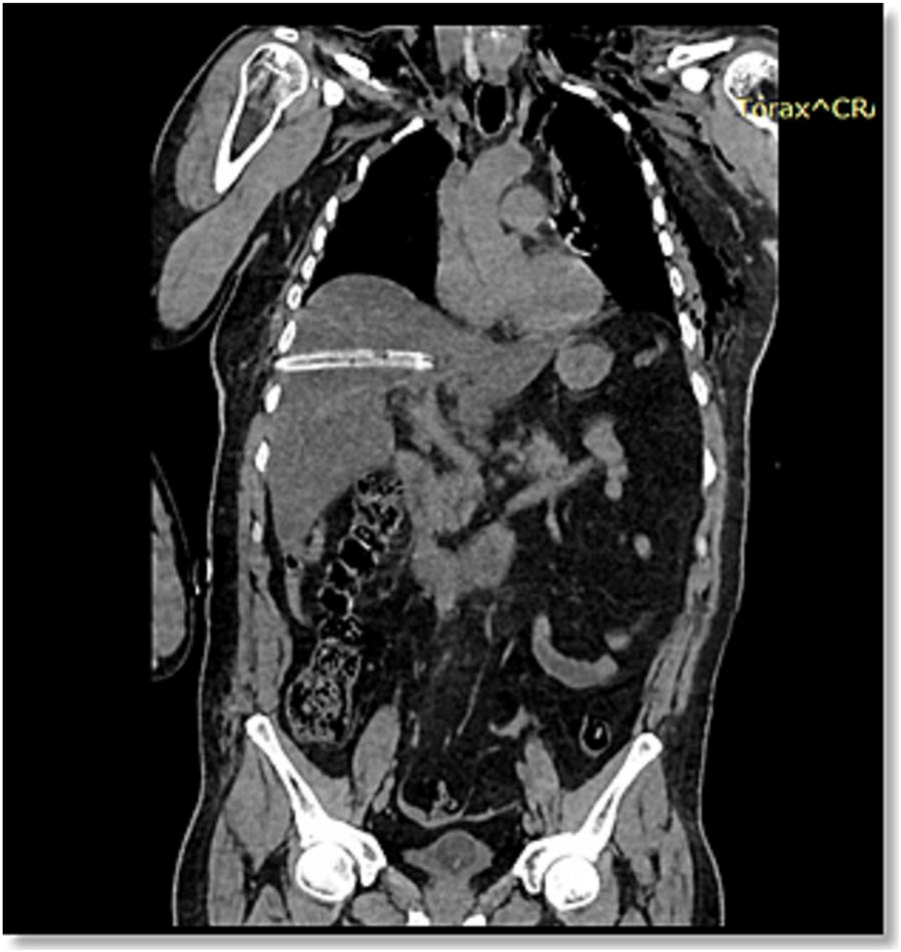
Non-contrast CT of the chest, abdomen, and pelvis, coronal view, demonstrating a chest tube inserted through the seventh intercostal space penetrating hepatic segments VII, VIII, and I. No free intraperitoneal fluid is identified.

**Figure 2:**
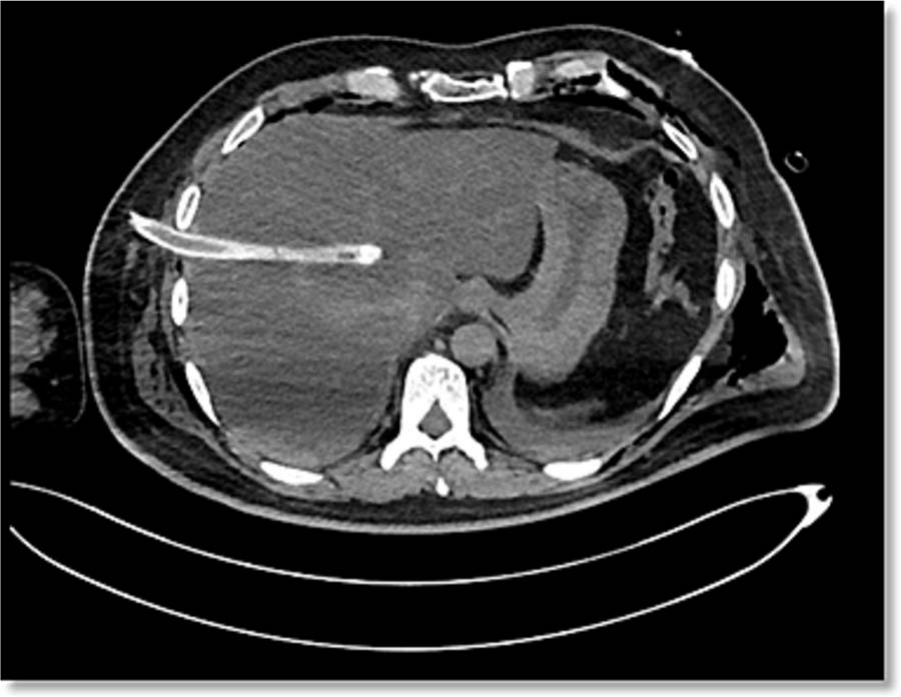
Non-contrast abdominal CT, axial view, demonstrating penetrating hepatic trauma caused by iatrogenic chest tube placement involving hepatic segments VII, VIII, and I.

After transfer to a tertiary center, thoracoabdominal computed tomography (CT) revealed that the right chest tube was inserted in the seventh intercostal space, demonstrating a parenchymal laceration involving hepatic segments VII, VIII, and I with a depth exceeding 3 cm, the injury was categorized as AAST-OIS (American Association for the Surgery of Trauma – Organ Injury Scale) Grade III and WSES (World Society of Emergency Surgery) Classification Grade II lesion (Figs 1 and 2). The patient was hemodynamically stable, FAST showed laminar fluid without significant hemoperitoneum, and CT excluded active extravasation or associated intra-abdominal injuries.

**Figure 3:**
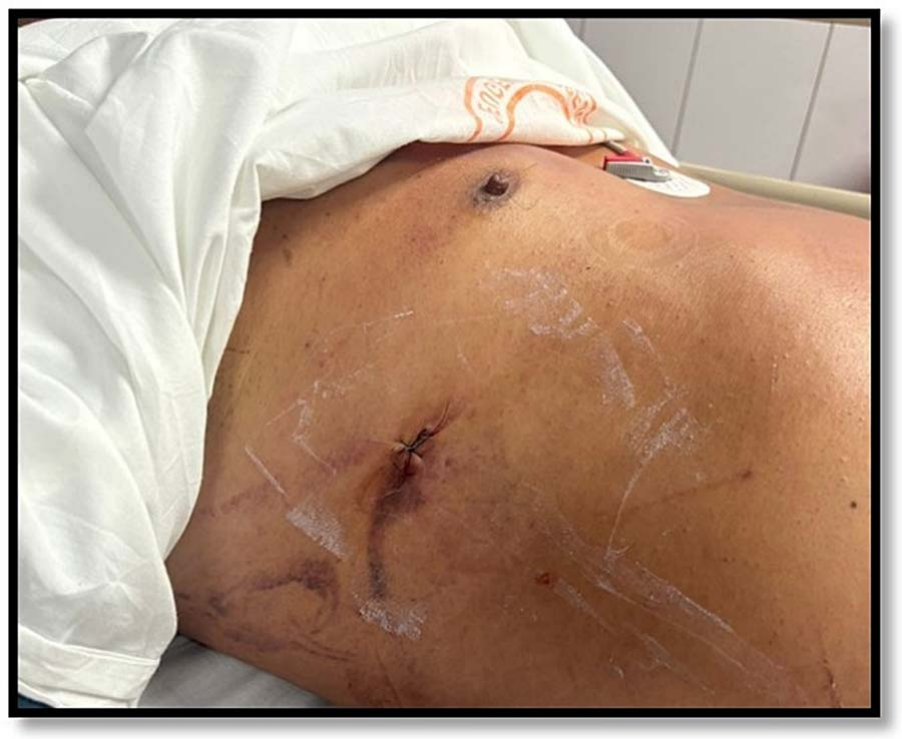
Wound after removal of the right chest tube.

**Figure 4:**
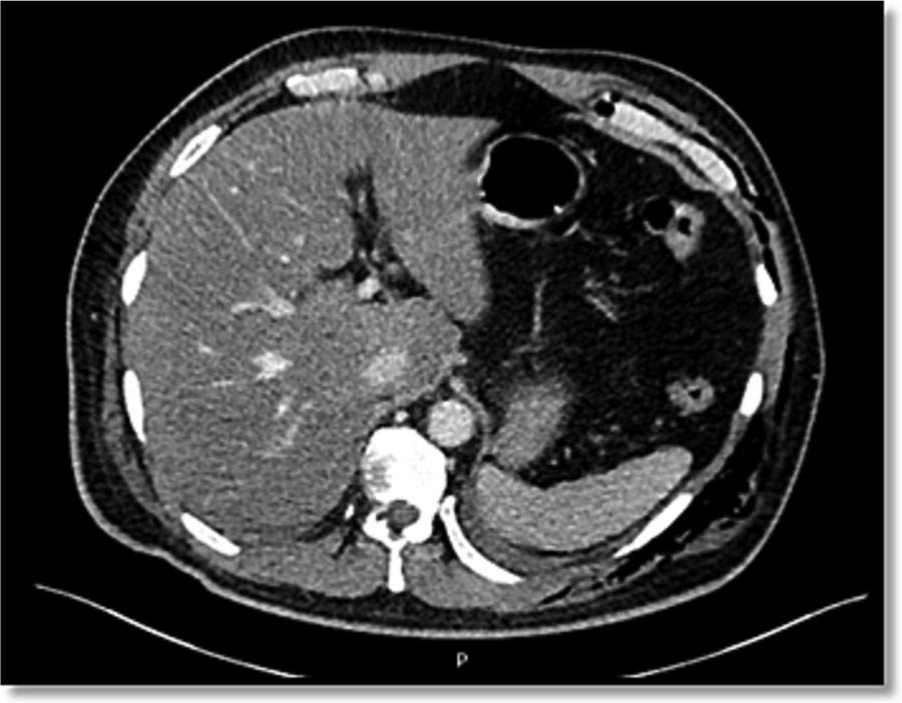
Triphasic abdominal CT, axial view, showing no evidence of hepatic parenchymal injury, preserved vascular integrity, and absence of free fluid or intraperitoneal air.

**Figure 5:**
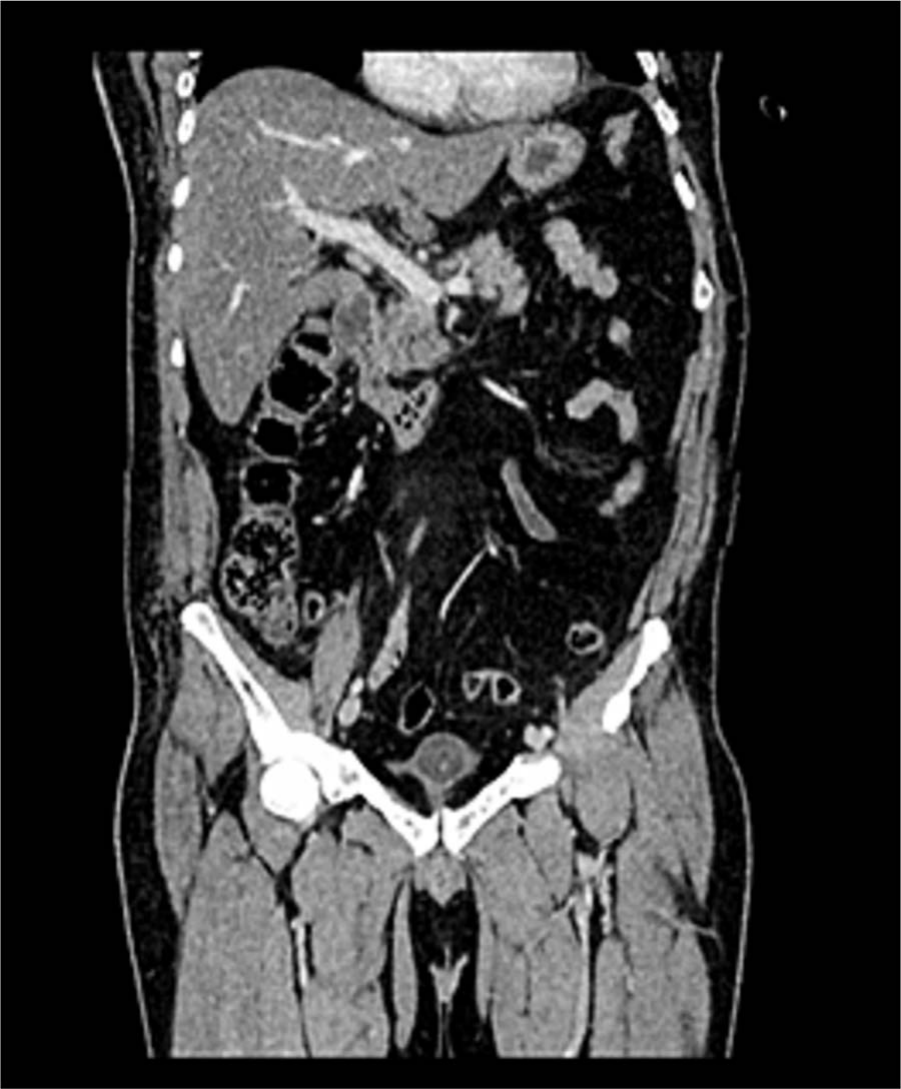
Triphasic abdominal CT, coronal view, demonstrating preserved integrity of the hepatic parenchyma.

Given these findings, NOM was selected. The chest tube was carefully withdrawn under continuous hemodynamic monitoring. The patient’s stability in this case allowed for direct extraction with full readiness for emergency laparotomy if required. Primary suture of the cutaneous tract was performed with 3–0 nylon to seal the entry site. No adjunctive hemostatic agents or catheter embolization were necessary, given the intrinsic hemostatic capacity of the hepatic parenchyma in the absence of major vascular injuries (Fig. 3). A triphasic CT at 12 hours demonstrated complete resolution of the hepatic lesion without parenchymal sequelae or free intraperitoneal fluid (Figs 4 and 5).

Due to thoracic instability resulting from multiple rib and sternal fractures, surgical stabilization and internal fixation was performed, achieving anatomical alignment, improved ventilatory mechanics, and adequate analgesia. The patient evolved favorably without hemorrhagic or respiratory complications and was discharged on postoperative day three with a referral for outpatient rehabilitation. Follow-up appointments were conducted at one and two months post-discharge, during which no late complications such as biliary fistulae, hepatic artery pseudoaneurysms, or cavitary collections were identified.

## Discussion

Chest tube insertion, though common, requires precise anatomical knowledge [4]. Complications may involve lung, heart, neurovascular bundle, diaphragm, spleen, intestine, and liver [1]. Hepatic injury from chest tube placement is extremely rare and lacks standardized management guidelines. Potential sequelae include subcapsular or intrahepatic hematoma, vascular or biliary injury, and hemoperitoneum. Clinical deterioration may manifest as abdominal distension, hypovolemic shock, or acute instability [3].

Bedside AP chest radiography is useful for initial assessment; however, CT remains the most sensitive and specific modality (96%–100%) for penetrating hepatic trauma [4]. NOM in penetrating hepatic trauma is appropriate in hemodynamically stable patients without peritoneal irritation or significant hemoperitoneum, provided intensive monitoring is available [4, 5].

In transhepatic chest tube malposition, management options include simple withdrawal, adjunctive hemostatic agents, or tract embolization, depending on bleeding risk [5, 8]. Removal should occur under radiologic guidance with readiness for urgent laparotomy if instability develops. Reported NOM success rates range from 85% to 94%. Delayed complications include pseudoaneurysm, abscess, hemobilia, and bilioma [9]. Hepatic pseudoaneurysm incidence ranges from 1.2% to 6.1%, with potential for rupture [10, 11]. There is no consensus regarding routine imaging surveillance [12, 13].

The 2020 WSES liver trauma guidelines classify AAST-OIS grade III injuries in stable patients as WSES grade II, recommending conservative management [14]. Bae *et al*. [9] reported liver injury in 10% of 137 thoracentesis/chest tube cases; 36% required endovascular therapy; and 29% surgical hemostasis. Angioembolization indications remain unclear. Yuan et al. [15] reported embolization failure up to 26.4%, reverting to conservative management.

The feasibility of non-operative management in this case was based on hemodynamic stability and the absence of signs of peritoneal irritation. In contrast to reports requiring urgent intervention such as injuries involving the inferior vena cava or the portal vein this case presented with limited hemorrhage and an absence of active extravasation on CT imaging.

## Conclusion

This study challenges the automatic classification of transhepatic chest tube malposition as a mandatory impalement trauma requiring laparotomy. Our findings demonstrate that a transhepatic trajectory, sparing hilar and retrohepatic structures, can be safely managed through controlled withdrawal and vigilant surveillance. This paradigm shift toward non-operative management optimizes resources and accelerates recovery. In hemodynamically stable patients, conservative protocols are feasible and essential to avoid unnecessary non-therapeutic laparotomies.
